# Human Chondrocyte Activation by Toxins From *Premolis semirufa*, an Amazon Rainforest Moth Caterpillar: Identifying an Osteoarthritis Signature

**DOI:** 10.3389/fimmu.2020.02191

**Published:** 2020-09-18

**Authors:** Isadora M. Villas-Boas, Giselle Pidde, Flavio Lichtenstein, Ana Tung Ching Ching, Inácio de Loiola Meirelles Junqueira-de-Azevedo, Carlos DeOcesano-Pereira, Carlos Eduardo Madureira Trufen, Ana Marisa Chudzinski-Tavassi, Kátia Luciano Pereira Morais, Denise V. Tambourgi

**Affiliations:** ^1^Immunochemistry Laboratory, Butantan Institute, São Paulo, Brazil; ^2^Centre of Excellence in New Target Discovery (CENTD), Butantan Institute, São Paulo, Brazil; ^3^Special Laboratory for Applied Toxinology, Butantan Institute/Center of Toxins, Immune-Response and Cell Signaling (CeTICS), São Paulo, Brazil

**Keywords:** osteoarthritis, toxins, caterpillar, chondrocyte, mediators, cell signaling

## Abstract

Pararamosis is a disease that occurs due to contact with the hairs of the larval stage of the Brazilian moth *Premolis semirufa*. Envenomation induces osteoarticular alterations with cartilage impairment that resembles joint synovitis. Thus, the toxic venom present in the caterpillar hairs interferes with the phenotype of the cells present in the joints, resulting in inflammation and promoting tissue injury. Therefore, to address the inflammatory mechanisms triggered by envenomation, we studied the effects of *P*. *semirufa* hair extract on human chondrocytes. We have selected for the investigation, cytokines, chemokines, matrix metalloproteinases (MMPs), complement components, eicosanoids, and extracellular matrix (ECM) components related to OA and RA. In addition, for measuring protein-coding mRNAs of some molecules associated with osteoarthritis (OA) and rheumatoid arthritis (RA), reverse transcription (RT) was performed followed by quantitative real-time PCR (RT-qPCR) and we performed the RNA-sequencing (RNA-seq) analysis of the chondrocytes transcriptome. In the supernatant of cell cultures treated with the extract, we observed increased IL-6, IL-8, MCP-1, prostaglandin E2, metalloproteinases (MMP-1, MMP-2, MMP-3 and MMP-13), and complement system components (C3, C4, and C5). We noticed a significant decrease in both aggrecan and type II collagen and an increase in HMGB1 protein in chondrocytes after extract treatment. RNA-seq analysis of the chondrocyte transcriptome allowed us to identify important pathways related to the inflammatory process of the disease, such as the inflammatory response, chemotaxis of immune cells and extracellular matrix (ECM) remodeling. Thus, these results suggest that components of *Premolis semirufa* hair have strong inflammatory potential and are able to induce cartilage degradation and ECM remodeling, promoting a disease with an osteoarthritis signature. Modulation of the signaling pathways that were identified as being involved in this pathology may be a promising approach to develop new therapeutic strategies for the control of pararamosis and other inflammatory joint diseases.

## Introduction

There are many venomous moth caterpillars from Order Lepidoptera that can cause severe injuries to humans. The reactions range from urticarial dermatitis, allergic reactions, renal failure, and osteochondritis to intracerebral bleeding (1). Among these venomous caterpillars, the Brazilian moth *Premolis semirufa* (Erebidae family), known as pararama in its larval stage, inhabits rubber plantations found in the Amazon forest and produces a singular clinical manifestation of envenomation (2–4). Pararamosis (pararama-associated phalangeal periarthritis) is a disease caused by contact with *P*. *semirufa* urticating hairs. This contact causes an intense itching sensation, followed by symptoms of acute inflammation. After repeated contact with this caterpillar, the inflammatory process becomes chronic, leading to joint immobility that is characterized by articular synovial membrane thickening with joint deformities (4–6).

Based on these clinical symptoms of pararamosis, previous studies by our group (7) showed that *P*. *semirufa* hair extract presents strong proteolytic activity and induces high antibody titer production and an intense inflammatory reaction in the tissues of inoculated mice that is characterized by the presence of macrophages and neutrophils. We also demonstrated using a murine model that the extract promotes activation of T lymphocytes and antigen-presenting cells and increased production of cytokines, such as IL-6, IL-10, IL-12, IL-17, and IL-23 (8). Moreover, the extract activates the alternative and lectin pathways of the complement system, generating biologically active fragments, such as C3a, C4a, and C5a anaphylatoxins in human serum, and direct cleavage of purified complement components such as C3, C4, and C5. These results led us to consider that the complement system plays a role in the inflammatory process seen in humans after envenomation by this caterpillar (9).

Thus, the disease caused in humans by contact with *P*. *semirufa* caterpillar hairs, in contrast to the manifestations observed due to exposure to other caterpillars, progresses to deformity by osteoarticular changes, with cartilage impairment, as observed in the clinical condition exhibited by joint diseases. The most common and best-investigated joint diseases are osteoarthritis (OA) and rheumatoid arthritis (RA) (10–12). Osteoarthritis is a multifactorial, chronic and degenerative disease of the joints that is characterized by progressive degradation of cartilage and bone damage, and the mechanisms that lead to it have largely been investigated. Chronic and excessive or repetitive mechanical loading of the articular cartilage produces hydrostatic and elastic stress and fluid flow, leading to alterations in chondrocyte morphology. These alterations induce expression of matrix metalloproteinases (MMPs) and disintegrin and metalloproteinase with thrombospondin motifs (ADAMTS), followed by proinflammatory cytokine production by synovial cells and chondrocytes (13, 14). In addition, endochondral ossification is a characteristic of osteoarthritis. Therefore, the imbalanced expression of MMPs and ADAMTSs play a central role in the first steps of OA, and inflammation is one of the first signals that persists during the whole process, as reviewed by Ripmeester et al. (15). OA progression leads to apoptosis and irreversible calcification of the cartilage matrix. In OA, some cytokines are seemingly produced by cartilage rather than synovial tissue (16, 17). The main pathological feature in the joints is cartilage degradation, accompanied by secondary synovitis (18).

In contrast, RA is a chronic, autoimmune, and systemic disease that affects the joints. This disorder results in synovial inflammation, hyperplasia within the inflammatory pannus, and bone erosion. These processes involve a complex network of interactions between innate and adaptive immunity (19). Additionally, RA is characterized by the production of rheumatoid factors and antibodies that are reactive to citrullinated proteins and by the progressive destruction of synovial and bone articular cartilage. The immune response and cartilage destruction include the production of many cytokines (20) and activation of effector cells and signaling pathways (21).

In view of the clinical similarities between pararamosis and joint diseases, we hypothesized that pararama venom components have the ability to interfere with the phenotype of cells present in the joints, such as chondrocytes, resulting in inflammation and promoting tissue injury. Therefore, to address the inflammatory mechanisms triggered by envenomation, we investigated the effect of *P*. *semirufa* hair extract on human chondrocytes by evaluating the production of cytokines, chemokines, MMPs, complement molecules, eicosanoids, and extracellular matrix (ECM) components related to OA and RA. In addition, we performed an RNA-sequencing (RNA-seq) analysis of the chondrocyte transcriptome, which allowed us to identify important pathways related to the inflammatory process of the pararamosis. Collectively, the chondrocyte molecular alterations observed after venom exposure indicate a phenotype signature in pararamosis that is similar to OA and RA. Modulation of the signaling pathways triggered by the caterpillar in human chondrocytes may be a promising approach for the treatment of pararamosis and other inflammatory joint diseases.

## Materials and Methods

### Preparation of *Premolis semirufa* Hair Extract

We collected caterpillars from *Premolis semirufa* Walker, 1856 (22) in areas of a rubber tree plantation of the city of São Francisco do Pará in Pará, Brazil {{Coord|1|10|08.7|S|47|47|26.3|W|}} and maintained them at the Immunochemistry Laboratory of the Butantan Institute in SP, Brazil. The Chico Mendes Institute for Biodiversity Conservation (ICMBIO) of the Brazilian Ministry of the Environment provided the license for capture, transportation, and maintenance of the animals (permission # 45166-4). Access to the venom was granted by the Brazilian Institute of the Environment and Renewable Natural Resources (IBAMA), an enforcement agency of the Brazilian Ministry of the Environment (010338/2014-4), and by the National System of Genetic Resource Management and Associated Traditional Knowledge (SisGen) (registration number A05C092). We carried out the extraction procedure of hair proteins and the determination of their enzymatic activity according to Villas-Boas and colleagues (7).

### Chondrocyte Culture and Cell Treatment With *P*. *semirufa* Hair Extract

Normal human articular chondrocytes that were derived from the knee (NHAC-kn) at the second passage were purchased from Lonza (Lonza Walkersville, Inc.) and cultured in chondrocyte growth medium (Lonza, Walkersville, MD, USA) containing 10% fetal bovine serum (FBS), growth factors and supplements [0.2% R3-insulin-like growth factor-1 (R3-IGF-1)], 0.5% human recombinant fibroblast growth factor-beta [hrFGF-β], 0.1% transferrin, 0.2% insulin, and 0.1% gentamicin/amphotericin-B [GA]-1000 at 37°C with 5% CO_2_, according to the manufacturer's instructions. Cells were grown in monolayer cultures, and the medium was changed every 2–3 days. For experiments, we used NHAC-kn at the 6th passage.

Cells were seeded into 96-well plates at a concentration of 5 × 10^4^ cells/mL and incubated at 37°C in an incubator with 5% CO_2_. After 24 h, we treated the cells with increasing concentrations of the extract (15, 30, and 60 μg/mL), corresponding to 0.3, 0.6, and 1.2 ng of protein/cell, in serum-free medium. We collected supernatants at 24, 48, and 72 h, centrifuged them at 400 × *g* at 4°C for 20 min, and aliquoted and froze the samples at −80°C for further analysis. As negative and positive controls, chondrocytes were cultured in the presence of phosphate buffered saline (PBS) or 10 ng/mL of interleukin-1 beta (IL-1β/IL-1F2, R&D System, code 201-LB-005), respectively.

### Cell Viability Analysis by MTT Assay

We assessed the viability of the attached cells by an MTT assay (23) based on the absorption of the MTT salt, 3- (4,5-dimethylthiazol-2-yl)−2,5-diphenyltetrazolium bromide (Invitrogen, Carlsbad, CA, USA). Viable cells (metabolically active) metabolize the MTT salt, and its reduction leads to insoluble formazan crystals accumulating in the cytoplasm.

### Analysis of Cytokines and Chemokines Produced by Chondrocytes

We assessed the concentration of cytokines and chemokines in chondrocyte culture supernatants by flow cytometry using the following BD Biosciences kits: BD™ Cytometric Bead Array (CBA) Human Inflammatory Cytokines, BD™ Cytometric Bead Array (CBA) Human Th1/Th2/Th17 Cytokine and BD™ Cytometric Bead Array (CBA) Human Chemokine. The assays were performed according to the manufacturer's recommendations. The samples were evaluated for the presence of the cytokines IL-1β, IL-6, IL-10, TNF, and IL-12p70, as well as for the chemokines IL-8, CCL5/RANTES, CXCL9/MIG, CCL2/MCP-1, and CXCL10/IP-10, and the concentration of each factor was determined using FCAP Array 3.0 software (BD Biosciences, San Jose, CA, USA).

### Detection of Prostaglandins, Leukotrienes, and Thromboxanes Produced by Chondrocytes

We assessed eicosanoid production by chondrocytes using the Prostaglandin E_2_ ELISA Kit – Monoclonal, Leukotriene B_4_ ELISA Kit and Thromboxane B_2_ ELISA Kit, according to the manufacturer's recommendations (Cayman Chemical, Ann Arbor, MI, USA). The concentration of each eicosanoid was determined according to the manufacturer's recommendations.

### Production of Components of the Complement System by Chondrocytes

The supernatants of the chondrocyte cultures were also analyzed for the secretion of complement components. We assessed the concentrations of C1q, C3, C4, C5, and C9 using Complement C1, Complement C3, Complement C4, Complement C5, and Complement C9 Human ELISA kits, according to the manufacturer's recommendations (Abcam, Cambridge, UK). In addition, we built a standard curve on the log-log graph to quantify the component concentrations, with the standard concentration listed on the x-axis and the absorbance on the y-axis.

### Evaluation of Matrix Metalloproteinases (MMPs) and Tissue Inhibitors of Metalloproteinases (TIMPs)

We analyzed the presence of MMPs and TIMPs in chondrocyte culture supernatants by using MMP1, MMP2, MMP3, MMP9, and MMP13 Human ELISA Kits and TIMP1 and TIMP2 Human Simple Step ELISA kits according to the manufacturer's recommendations (Abcam, Cambridge, UK). Moreover, we built a standard curve on the log-log graph to dose each MMP concentration, with the standard concentration shown on the x-axis and the absorbance on the y-axis.

### Evaluation of the Presence of Aggrecanase-1 (ADAM-TS4)

We assessed the presence of aggrecanase-1 in the supernatants of chondrocyte cultures by using the Sensitive Aggrecanase Activity Assay kit, following the manufacturer's recommendations (BioTeZ, Berlin, Germany).

### Analysis of the Expression of Aggrecan, Type II Collagen and HMGB1 by High-Content Screening (HCS)

Normal human chondrocytes (NHAC-kn) at the 6th passage were cultured in 96-well microplates (Greiner Bio-One, 655986) at a density of 8 × 10^3^ cells/well in chondrogenic growth medium containing supplements and growth factors (Lonza, Walkersville, MD, USA) at 37°C and 5% CO_2_. After 24 h, the cells were treated with increasing concentrations of extract at 12 and 49 μg/mL, corresponding to 0.3 and 1.2 ng of protein/cell, in serum-free medium for 24, 48, and 72 h. These concentrations were calculated based on the quantity of extract (μg) *per* cell and were used throughout all 96-well plate experiments. As negative and positive controls, we cultured chondrocytes in the presence of PBS or 8 ng/mL interleukin-1 beta (IL-1β), respectively.

After the treatments, the cultures were washed with PHEM buffer (2 mM HEPES, 10 mM EGTA, 2 mM MgCl_2_, 60 mM PIPES pH 6.9) and fixed for 1 h with cold 4% PFA. The cells were permeabilized with 0.1% Triton X 100 for 5 min, blocked with 1% bovine serum albumin (BSA) for 30 min, and then incubated with primary antibody overnight at 4°C. After washing with PHEM glycine (3×), the cells were incubated with the fluorescent dye at room temperature for 1 h, and the plates were subjected to high content imaging analysis by using MetaXpress High Content Image Acquisition & Analysis Software (Molecular Devices). The primary antibodies used were anti-Aggrecan (Abcam plc, Cambridge, UK) diluted 1:100, anti-collagen II (Abcam plc, Cambridge, UK) diluted 1:100, and anti-HMGB1 (Santa Cruz Biotechnology, Inc., CA, USA) diluted 1:500 and were incubated overnight at 4°C. After washing with PHEM (3×), the cells were incubated with Alexa Fluor 647 goat anti-rabbit and Alexa Fluor 488 rabbit anti-mouse secondary antibodies (Life Technologies, Camarillo, CA, USA) at a 1:1,000 dilution for 1 h at room temperature in the dark. The cells were washed with PHEM (3×), nuclei were counted using Hoechst 33342 (5 μM, Life Technologies, Thermo Fisher Scientific) staining for 1 h, and the stained samples were subjected to high content imaging analysis. The image acquisition and fluorescence intensity measurements were conducted by automatic scanning using MetaXpress software with a 10× objective. For each treatment condition and channel, nine images *per* well, in triplicate, were acquired and analyzed. MetaXpress software (Molecular Devices, Sunnyvale, CA, USA) was used to calculate the stained area using the Custom Module and the fluorescence intensity was calculated using the MultiWaveScoring module.

### Transcriptomic Analysis

#### RNA Isolation

Cells were seeded into 24-well plates at a density of 1 × 10^5^ cells/well in chondrocyte growth medium containing supplements and growth factors (Lonza, Walkersville, MD, USA) and incubated at 37°C and 5% CO_2_. After 24 h, the chondrocytes were maintained in serum-free medium with the highest concentration of *P*. *semirufa* hair extract (60 μg/mL *per* well) for 24 h. In parallel, chondrocytes were maintained in the presence of the same volume of PBS or 10 ng/mL of interleukin 1 beta (IL-1β) as negative and positive controls, respectively. At the end of the treatment period, the growth media was removed, and total RNA was isolated from the cell cultures (total of 1 × 10^6^ cells *per* treatment, in triplicate) by using TRIzol (Life Technologies, Inc., Camarillo, CA, USA) according to the manufacturer's protocol. RNA samples were visualized with an agarose gel, and their concentration was assessed on a Nanodrop 2000c spectrophotometer. The Agilent 2100 Bioanalyzer (RNA 6000 Nano LabChip, Agilent Technologies, Santa Clara, CA, USA) was used to determine the RNA integrity number (RIN). All RNA samples had a RIN > 9.10.

#### Library Preparation and Sequencing

The messenger RNAs (mRNAs) were purified from the total RNA isolated from the human chondrocyte cultures and used to prepare complementary DNA (cDNA) libraries following the protocol of the *TruSeq RNA Sample Prep Kit V2* (Illumina, San Diego, CA, USA). Briefly, mRNAs were isolated with dT-oligos, purified, and fragmented by heating at 94°C (4 min) in the kit fragmentation buffer. Double-stranded cDNAs were synthesized, end-repaired and A-tailed. Sequencing adapters were then ligated to the cDNA fragments according to the manufacturer's protocol. The cDNA fragments were enriched by 15 cycles of PCR amplification. The quality of the libraries was evaluated by cDNA size distribution, as measured by a 2100 Bioanalyzer with DNA1000 assay (Agilent Technologies, Santa Clara, CA, USA). An ABI StepOnePlus Real-Time PCR System was used to estimate the size of the libraries before sequencing. The cDNA libraries were sequenced on an Illumina HiSeq 1500 System in Rapid Run mode using a paired-end flow cell with a 2^*^101 bp paired-end configuration.

#### Quality and Filtering FASTQ Reads

The raw sequencing read contaminants were removed with Bowtie version 2 2.2.5 (24), and Trimmomatic version 0.36 was used to trim and remove reads with low-complexity and homopolymer enriched regions, poly-A/T/N tails, adapter sequences and low-quality bases. Reads were filtered out if more than 90% of them corresponded to a homopolymer or low-complexity regions and if the mean quality score was lower than 25 in a window size equal to 15. After trimming, all reads smaller than 40 bp were discarded. A quality check was performed using FastQC. Next, Hisat2 (25) was used to align reads from each sample against the human reference genome (annotation version 92), generating the count values for the genes that were used in the differential expression analysis, which were further described.

The read quality results using FastQC and map quality plots indicated that the lowest covered library had more than 20 million reads and that the percentages of mapped reads were higher than 90% for all libraries. We also compared the LFC (log2-fold change) of RNA-seq and RT-qPCR for Ext x Ctrl and IL1B x Ctrl (further described). For Ext x Ctrl, the correlation was ~84%, and for IL1B x Ctrl, the correlation was ~89%. This accuracy shows the high quality of the RNA extraction, the Illumina Rapid Run sequencing and the mathematical model.

#### cDNA Synthesis and RT-qPCR

For measuring protein-coding mRNAs, reverse transcription (RT) was performed using SuperScript III according to the manufacturer's instructions (Thermo Fisher Scientific, Waltham, MA, USA) followed by quantitative real-time PCR (RT-qPCR). For all genes, oligo-dT primer reverse transcription was performed using 350 ng of total RNA isolated from the human chondrocytes in a 20 μL RT reaction with SuperScript III, followed by qRT-PCR using 5 μL of 8-fold diluted RT reaction in 20 μL of qRT-PCR (ViiA 7 Real-Time PCR System, Thermo Fisher Scientific, Waltham, MA, USA). Transcript levels were normalized to glyceraldehyde-3-phosphate dehydrogenase (*GAPDH*), and the results are presented as the relative abundance using the 2–ΔΔCT method (26). The primer sequences are listed in Supplementary Table 1.

### Bioinformatics and Systems Biology

#### Expression Analysis

RNA-seq *in silico* analysis included diverse quality and quantity steps to assess transcriptome expression (Supplementary Figure 1). We assessed read quality using FastQC version 0.11.5. After the quality procedures, we used Ensembl Gene ID transcripts to map the reference genome (GRCh38) (annotation version 92). To quantify transcripts, we used featureCounts (27) from subread version 1.6.2, resulting in a table of 58,233 gene IDs as rows, with samples in columns and cells as the raw read counts. Then, we removed genes with low expression, i.e., row sum of expressions < 1, resulting in 18,671 valid transcripts. The data generated for this study were deposited at the Sequence Read Archive (SRA) under SRA accession number PRJNA592966.

By using edgeR version 3.26.8, we calculated the normalized expression table in “counts *per* million” (CPM), which was input to infer the differentially expressed genes (DEGs), which were defined as an absolute value of log2-fold change between two groups greater than one and a false discovery rate (FDR) <0.05. For that, we only compared (a) Ext × Ctrl and (b) IL1B × Ctrl.

One approach that defined the experiment's success was evaluating data clustering using the multidimensional scaling plot (MDS). The main idea was to verify whether the samples clustered well as experimental groups. We observed that the MDS plot showed accurate clustering among the control, extract and IL-1β treatments (data not shown).

#### Enrichment Analysis

We performed an enrichment analysis with all recognized DEGs from the extract treatment vs. the control comparison. For that, we used two different techniques: (1) Gene Set Enrichment Analysis (GSEA), based on Kolmogorov-Smirnov statistics, and used fast-GSEA (fGSEA version 1.10.1) to calculate Pathway Enrichment Analysis (PEA) and (2) Over-Representation Analysis (ORA) using String-db (version 11.0)/KEGG and MetaCore™ (version 6.36 build 69400/2018). We also used MetaCore to calculate Maps (pathways) and Network Statistics, in addition to other methods. The FDR cutoff was set to 0.05 for each technique.

In the present work, we focused our analysis on genes that are associated with OA and evaluated expression of these factors in the supernatant or in the extracellular matrix of chondrocyte cultures. The preselected genes were *ACAN, ADAMTS4, BGN, C1QA, C1QB, C1R, C1S, C2, C3, C4A, C4B, C5, C7, C8A, C8B, C9, CCL2, COL1A1, COL2A1, CXCL8, GJA1, GJC1, HAS2, HAS3, HMGB1, HYAL1, HYAL2, HYAL3, IL18, IL1A, IL1B, IL6, KRT19, MMP1, MMP2, MMP3, MMP9, MMP13, PTGES, PTGES2, SOX9, TGFB1, TIMP1, TIMP2, TNF*, and *TP53*.

#### Statistical Analysis

All reported experiments were performed independently at least twice, and the data are expressed as the mean ± SEM. Statistical comparisons for wet-laboratory experiments were calculated using Student's *t*-test or two-way ANOVA followed by Dunnett *post hoc* tests. For these statistical calculations, we used GraphPad Prism-7 (San Diego, CA, USA) and considered a *p*-value < 0.05 to be significant.

## Results

### Human Chondrocyte Activation by *P*. *semirufa* Hair Extract: Production of Cytokines, Chemokines and Prostaglandin E_2_

**Figure 1:**
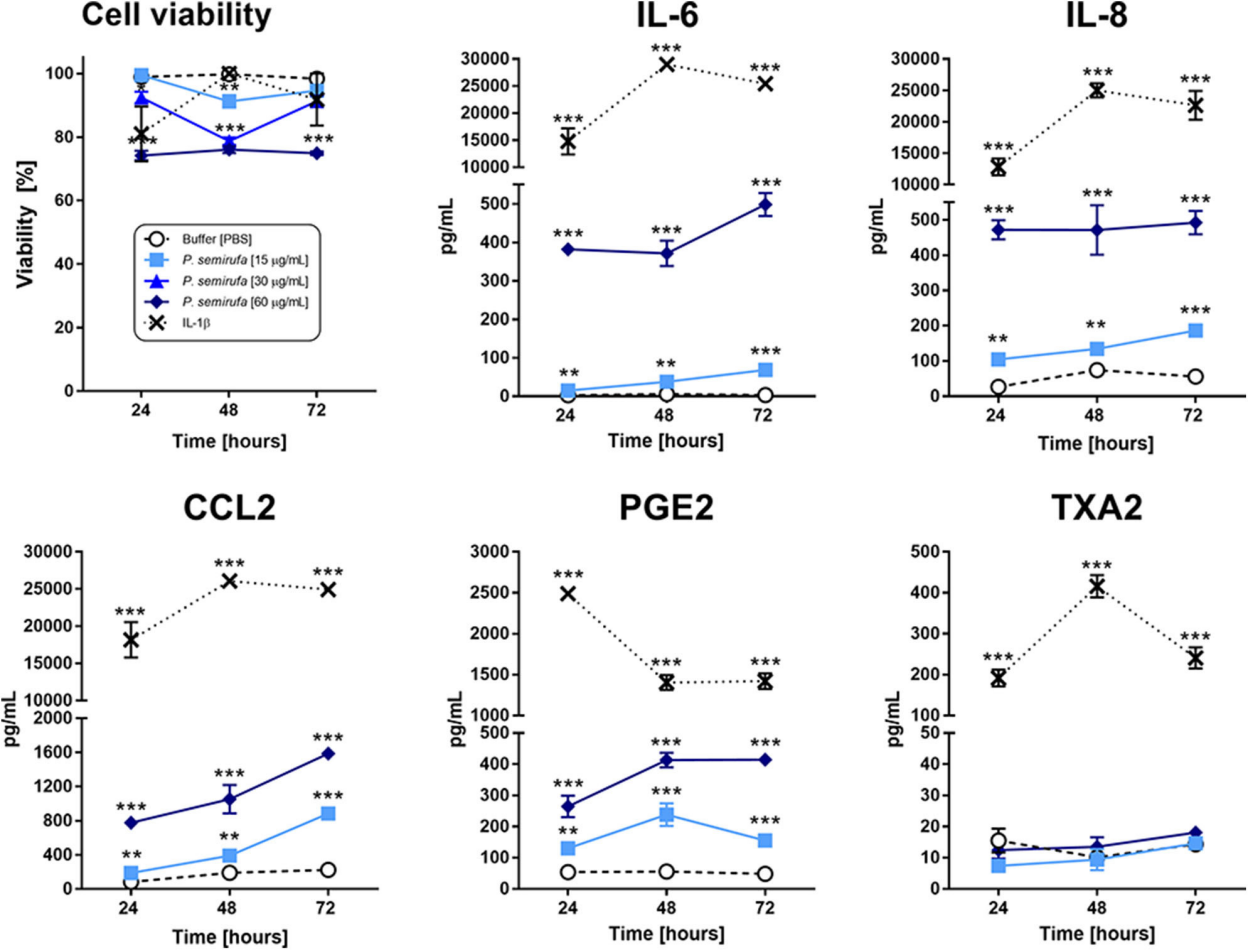
Assessment of cytokines, chemokines, and eicosanoids in chondrocytes treated with the *P*. *semirufa* hair extract. Chondrocytes were cultured in 96-well plates at a density of 5 × 10^4^ cells/mL and treated with buffer, IL-1β (10 ng/mL) or pararama hair extract (15, 30, or 60 μg/mL) for 24, 48, and 72 h. After each treatment period, we measured the cell viability by an MTT assay. The supernatants were collected from cells treated with buffer 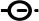, IL-1β [-x-], 15 μg/mL 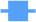 or 60 μg/mL 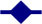 pararama hair extract for 24, 48, and 72 h by centrifugation at 400 ×g at 4°C for 20 min to assess the concentration of cytokines, chemokines, and eicosanoids. The results represent two separate experiments performed in duplicate and are expressed as the mean of the concentrations of the molecules ± SEM. The data were analyzed using two-way ANOVA and Dunnett's *post hoc* test. **p < 0.01; ***p < 0.001 vs. the control (buffer treatment).

To assess the effect of *P*. *semirufa* hair extract on human chondrocytes, we analyzed the viability of these cells after treatment with three extract concentrations (15, 30, and 60 μg/mL) for 24, 48, and 72 h. Figure 1 shows that the lowest extract concentrations (15 and 30 μg/mL) induced a small reduction in cell viability (9 and 11%, respectively) after 48 h of incubation. After 24 h, the highest concentration (60 μg/mL) induced a reduction of ~25% in cell viability. Based on these results, we conducted the subsequent experiments using the extract at 15 and 60 μg/mL. The positive control, IL-1β, induced a reduction in cell viability of ~20% or less over time.

Inflammatory mediator production is an essential event in the progression of joint diseases and possibly in pararamosis. Thus, we evaluated cytokines, chemokines, and eicosanoids in the supernatants of chondrocytes treated with pararama hair extract. Figure 1 shows that only IL-6, IL-8, and MCP-1 were significantly induced in a dose- and time-dependent manner in cell cultures treated with the extract compared to those of the negative control (buffer). The positive control IL-1β was used to mimic the pathophysiology of joint inflammation and induced the production of the same cytokines and chemokines but at increased concentrations.

Figure 1 also shows that only the extract induced the production of prostaglandin E_2_ (PGE2). In the positive control (IL-1β), thromboxane A_2_ (TXA2) and prostaglandin were detected in the supernatants, and PGE_2_ was produced at higher levels than that of the extract. LTB4 was not detected in these treatment conditions (data not shown).

### Pararama Hair Extract Induces Chondrocytes to Produce Complement System Components

**Figure 2:**
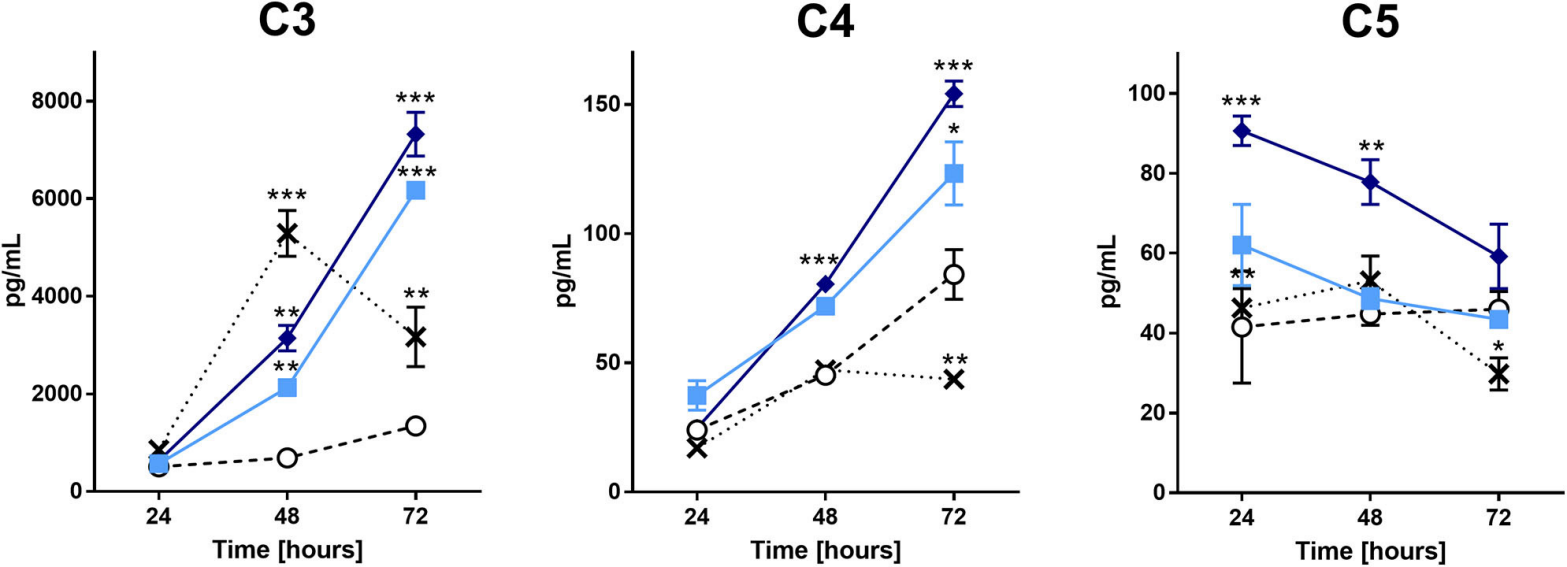
Complement components analysis in the supernatant of chondrocytes treated with the *P*. *semirufa* hair extract. Chondrocytes were cultured in 96-well plates at a density of 5 × 10^4^ cells/mL and treated with buffer 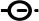, IL-1β [-x-], 15 μg/mL 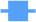 or 60 μg/mL 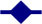 pararama hair extract for 24, 48, and 72 h. After each treatment period, we removed the supernatants, centrifuged them at 400 ×g at 4°C for 20 min, and assessed the concentrations of complement components by ELISA. The results represent two separate experiments performed in duplicate and are expressed as the mean of the concentrations of the complement components ± SEM. The data were analyzed using two-way ANOVA and Dunnett's *post hoc* test. *p < 0.05; **p < 0.01; ***p < 0.001 vs. the control (buffer treatment).

Considering the importance of the complement system in the inflammatory process, we evaluated the levels of C1q, C3, C4, C5, and C9 in the supernatants of chondrocyte cultures treated with the extract. Figure 2 shows that the production of C3, C4, and C5 components was significantly higher in cells treated with the extract than in the positive and negative controls (IL-1β and buffer, respectively). Interestingly, the extract treatment reduced the C5 component concentration over time. There was no increase in the production of C1q or C9 by the treated cells (data not shown).

### Pararama Hair Extract Induces Chondrocytes to Produce Molecules That Act on the Extracellular Matrix

**Figure 3:**
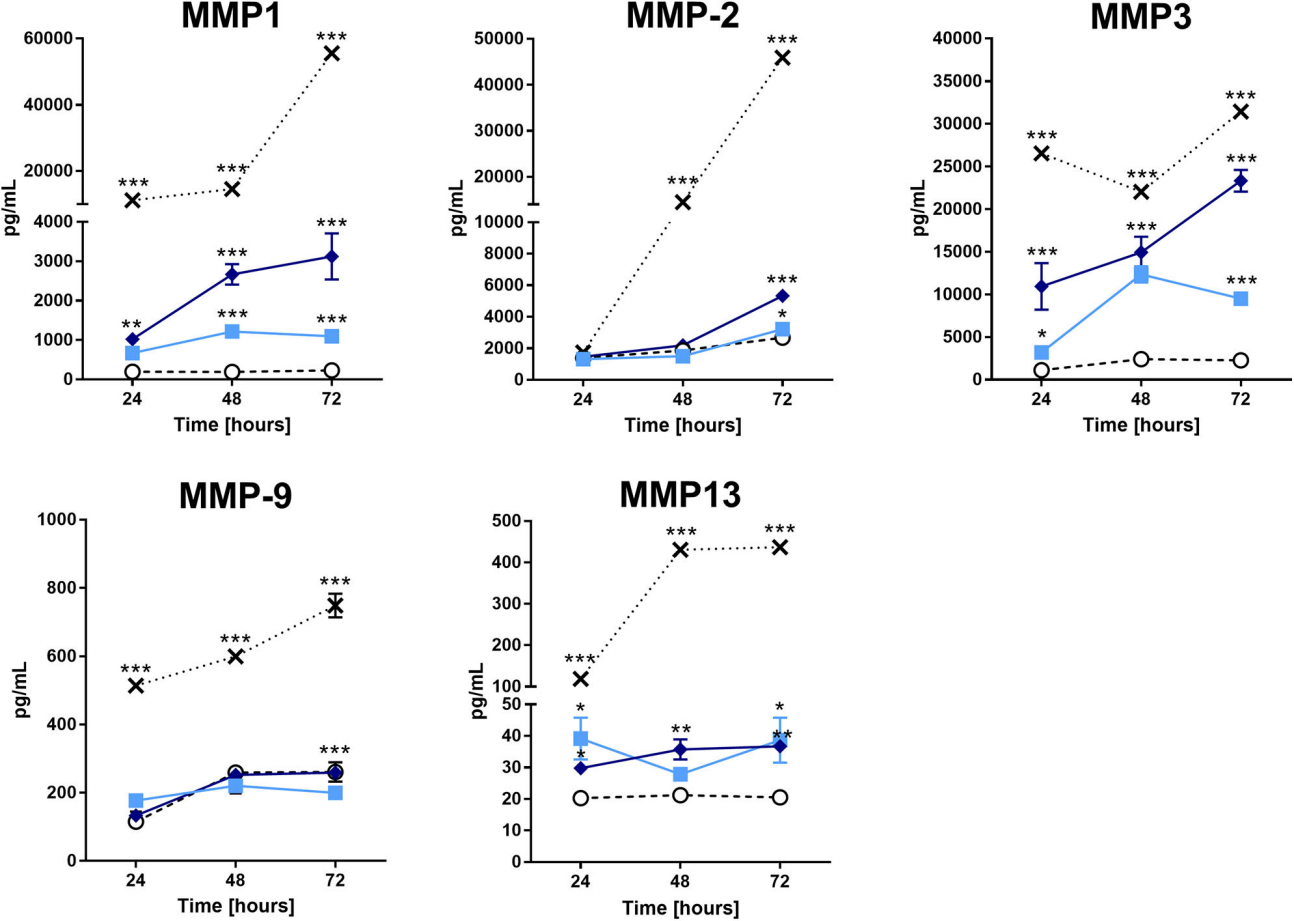
Matrix metalloproteinases analysis in the supernatant of chondrocytes treated with the *P*. *semirufa* hair extract. Chondrocytes were cultured in 96-well plates at a density of 5 × 10^4^ cells/mL and treated with buffer 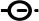, IL-1β [-x-], 15 μg/mL 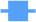 or 60 μg/mL 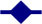 pararama hair extract for 24, 48, and 72 h. After each treatment period, we removed the supernatants, centrifuged them at 400 ×g at 4°C for 20 min, and assessed the concentration of matrix metalloproteinases by ELISA. The results represent two separate experiments performed in duplicate and are expressed as the mean of the concentrations of the metalloproteinases ± SEM. The data were analyzed using two-way ANOVA and Dunnett's *post hoc* test. *p < 0.05; **p < 0.01; ***p < 0.001 vs. the control (buffer treatment).

As mentioned before, MMPs and ADAMTSs are capable of degrading several matrix components, as well as type II collagen. Therefore, we assessed MMP production in the supernatants of the cultures. Figure 3 shows a significant increase in MMP-1, MMP-2, MMP-3, and MMP-13 in the supernatants of chondrocytes treated with the extract compared to those of the buffer treatment. The positive control (IL-1β) induced an increase in the tested MMPs. On the other hand, we did not detect any increase in aggrecanase (ADAMTS4) activity or tissue inhibitor of metalloproteinases (TIMPs) in the supernatants of the pararama hair extract- and IL-1β-treated cells compared with those of the buffer-treated cells (data not shown).

### Pararama Hair Extract Reduces the Expression of Aggrecan and Type II Collagen and Increases HMGB1 in Chondrocytes

**Figure 4:**
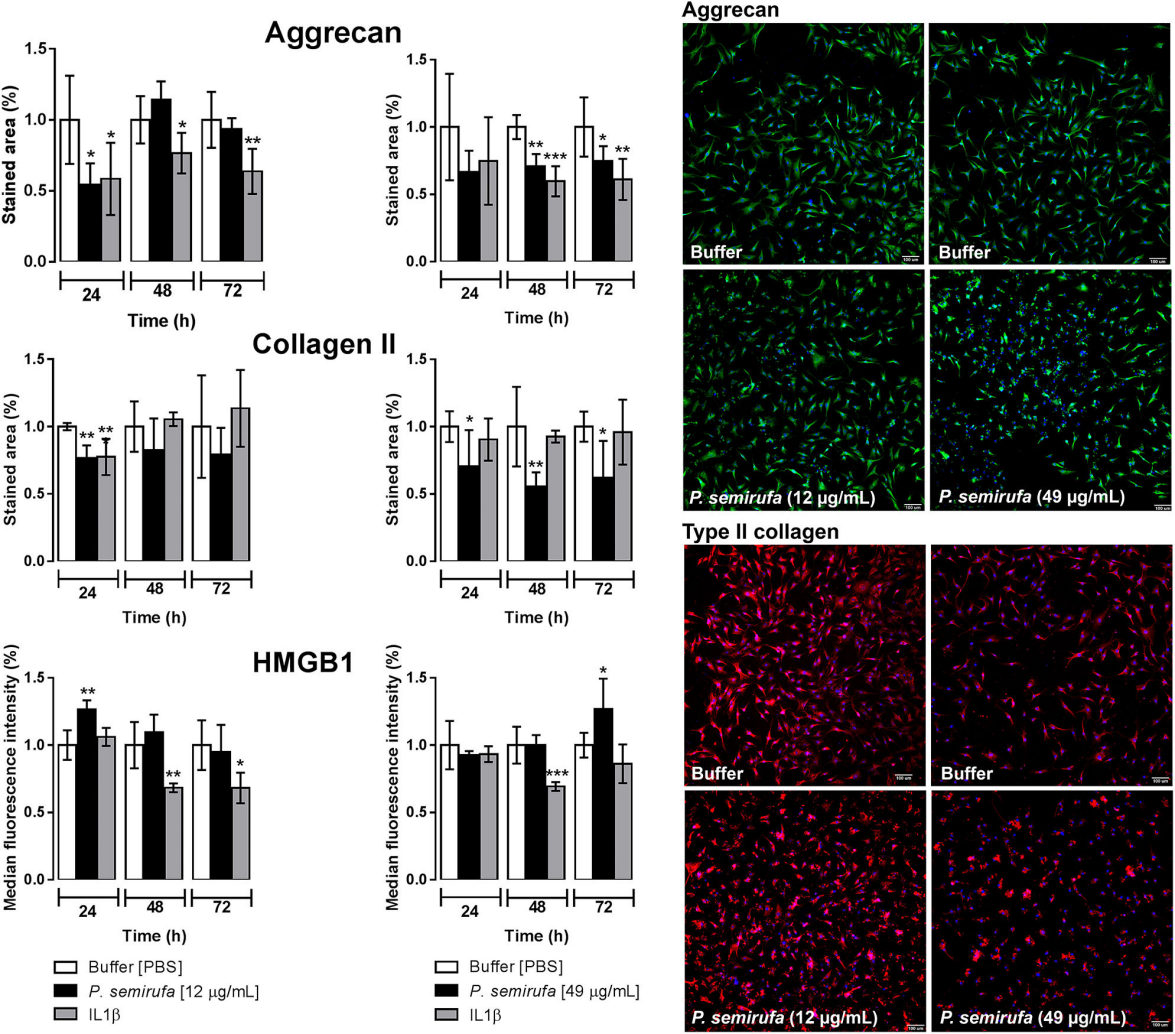
Evaluation of aggrecan and type II collagen production and HMGB1 expression by high-content screening (HCS). Chondrocytes were cultured in 96-well plates at a density of 4 × 10^4^ cells/mL and treated with buffer, IL-1β or pararama hair extract (12 and 49 μg/mL) for 24, 48, and 72 h. After each treatment period, the cells were fixed and blocked. Then, the cells were incubated with anti-Aggrecan, anti-collagen type II or anti-HMGB1 antibodies. In parallel, the cell counts were assessed using Hoechst 33342 staining. The image acquisition and fluorescence intensity measurements were conducted by automatic scanning by using MetaXpress software and a 10× objective, with the Custom Module to calculate the stained area and Multi Wave Scoring Module. For each condition and channel, nine images per well in triplicate were acquired and analyzed. Representative fluorescence microscopy images correspond to the cells obtained after 24 h of treatment. The results were normalized and represent two independent experiments performed in triplicate and are expressed as the mean of the stained area ± SEM or the mean of the median fluorescence intensity ± SEM. The data were analyzed using Student's *t*-test. *p < 0.05; **p < 0.01; ***p < 0.001 vs. the control (buffer treatment).

The integrity of both Aggrecan and type II Collagen is important in the structure of healthy cartilage. Therefore, we assessed their presence by a high-content screening (HCS) to evaluate the effects of the extract on these molecules in human chondrocytes. In parallel, we also investigated the presence of high mobility group box 1 (HMGB1), a protein that is associated with inflammatory diseases, such as RA and OA. Figure 4 shows a reduction in the stained area for both Aggrecan and type II Collagen in cells treated with the extract compared to that of buffer treatment. The Aggrecan reduction was more pronounced after 24 h of treatment, whereas we observed the reduction in type II Collagen at each time point. For HMGB1 protein, there was an increase in the fluorescence intensity within the nucleus after 24 and 72 h of extract treatment.

### RT-qPCR Analysis of Chondrocytes Treated With Pararama Hair Extract

**Figure 5:**
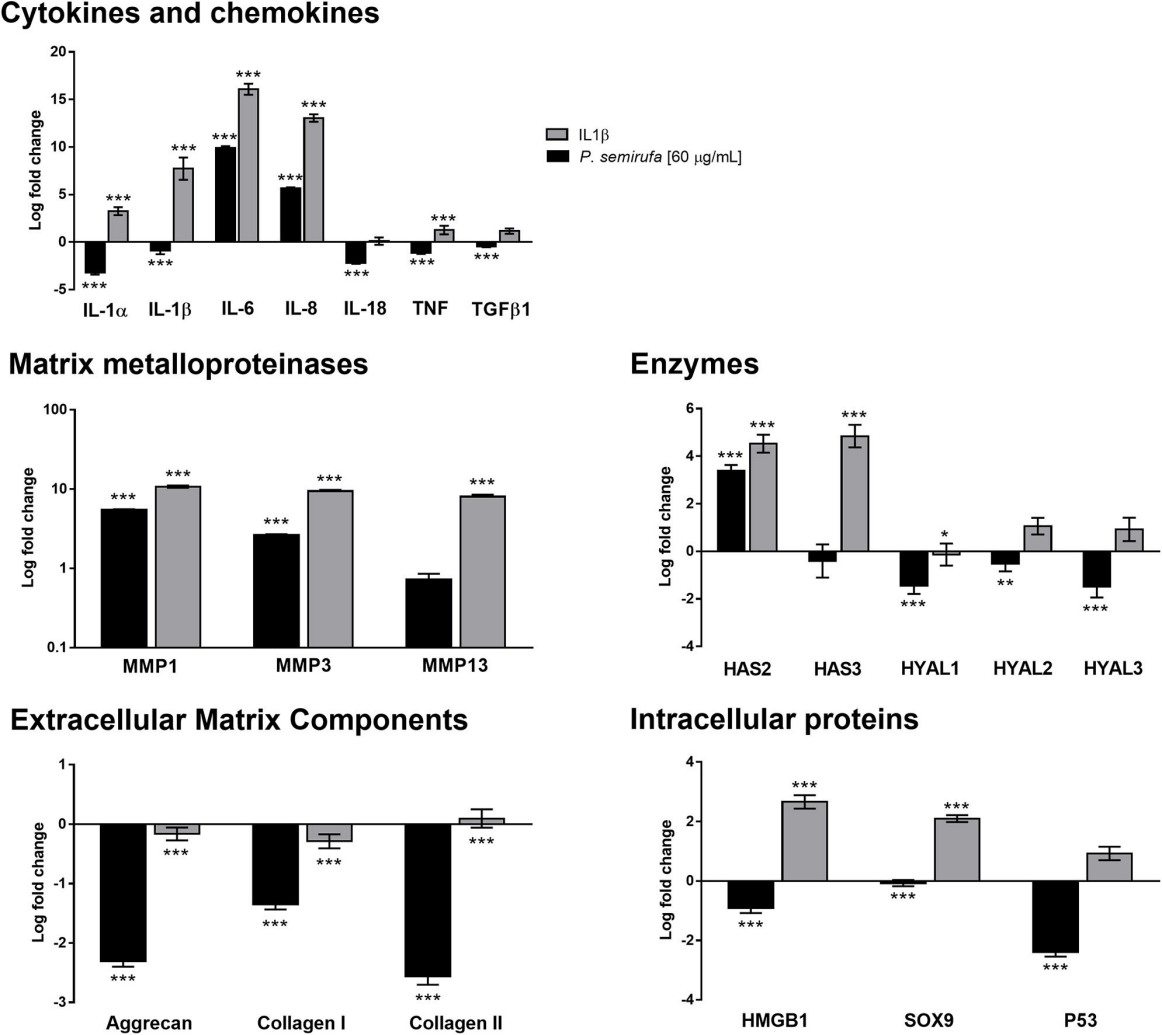
Gene expression profile in chondrocytes treated with the *P*. *semirufa* hair extract. Chondrocytes (1 × 10^5^ cells/well) treated with buffer, IL-1β or extract (60 μg/mL) were collected, and total RNA was extracted using TRIzol. Relative mRNA quantification was carried out by RT-qPCR. All experiments were performed in triplicate, and the values are presented as the mean ± SEM normalized to *GAPDH* as an endogenous control. The data were analyzed using Student's *t*-test. *p < 0.05; **p < 0.01; ***p < 0.001 vs. the control, which was arbitrarily set to 1 (buffer treatment).

In this study, we performed quantitative real-time PCR (RT-qPCR) to investigate the gene expression profile of some molecules associated with OA and RA (Supplementary Table 1). The gene expression results, shown in Figure 5, are consistent with the results from other experiments, in which we observed an increase in IL-6 and IL-8 in supernatants and a lack of IL-1β following the extract treatment. *IL-1*α and *IL-1*β genes were downregulated following extract treatment and were upregulated by IL-1β treatment; however, *IL-6* and *IL-8* were both upregulated following extract and IL-1β treatments. In addition, *IL-18, TNF*, and *TGF-*β*1* gene expression was downregulated in chondrocytes after extract treatment (Figure 5).

*MMP-1* and *MMP-3* were upregulated and highly expressed following both extract and IL-1β treatment, with *MMP-13* only upregulated in cells that were treated with IL-1β (Figure 5). These results showed a positive correlation with the protein data obtained in IL-1β- and pararama-treated cell supernatants.

The hyaluronan synthase 2 *(HAS2*) gene was upregulated following both extract and IL-1β treatments; however, *HAS3* was not changed by treatment with the extract but was upregulated by IL-1β treatment. In addition, hyaluronidase (HYAL), *HYAL-1, HYAL-2* and *HYAL-3* genes were downregulated following the extract treatment but were not modulated in the positive control group (Figure 5).

Gene expression analysis showed a reduction in the expression of some inflammatory joint disease markers, such as Aggrecan and types I and II Collagen, after extract and IL-1β treatment. Genes related to intracellular proteins, such as *HMGB1* and *SOX9*, were downregulated following extract treatment but were upregulated in the positive control. However, *P53* showed a distinct behavior; it was downregulated by the extract but was not altered by IL-1β treatment (Figure 5).

### Transcriptome Analysis of Human Chondrocytes Treated With Pararama Hair Extract

In this study, we performed transcriptomic analysis to elucidate the response of human chondrocytes to *P*. *semirufa* hair extract treatment after 24 h of *in vitro* stimulation. We used edgeR to normalize the gene expression in “counts per million” (CPM) over the 18,671 valid transcripts and to calculate the differentially expressed genes (DEGs), and here we show only two comparisons: “extract treatment x buffer treatment” (Ext × Ctrl) and “IL-1β treatment × buffer treatment” (IL-1β × Ctrl). There were 3,553 DEGs in the Ext × Ctrl group, of which 1,583 were upregulated and 1,970 were downregulated, and 5,506 DEGs in the IL-1β × Ctrl group, of which 2,601 were upregulated and 2,905 were downregulated.

The gene expression analysis focused on chondrocyte molecules that were predicted to be found in the culture supernatants, on the extracellular matrix or on those genes evaluated by RT-qPCR analyses, totaling 46 selected genes. A broader whole transcriptome data analysis remains to be explored and will be the scope of a future study.

Thus, 13 out of the 46 selected genes were DEGs: *ACAN, C3, CCL2, CXCL8, GJA1, HAS2, IL6, MMP1, MMP2, MMP3, MMP13, PTGES*, and *SOX9*. We did not detect the transcription of genes coding for *C1QA, C1QB, C2, C7, C8A, C8B, C9*, or *IL18* in the control, extract, or IL-1β treatment groups. We observed the transcription of some other genes, such as *ADAMTS4, BGN, C1R, C1S, C4A, C4B, C5, COL1A1, COL2A1, GJC1, HAS3, HMGB1, HYAL1, HYAL2, HYAL3, IL1A, IL1B, KRT19, MMP9, PTGES2, TGFB1, TIMP1, TIMP2, TNF*, and *TP53*, but they were not DEGs (Supplementary Table 2).

Supplementary Figure 2 shows a heatmap of the selected transcribed genes, in which the hierarchical clustering analysis shows consistent grouping among the treatments. In addition, although IL-1β treatment showed more upregulated genes compared to those of extract treatment, we can see that the response to these treatments was very similar.

**Figure 6:**
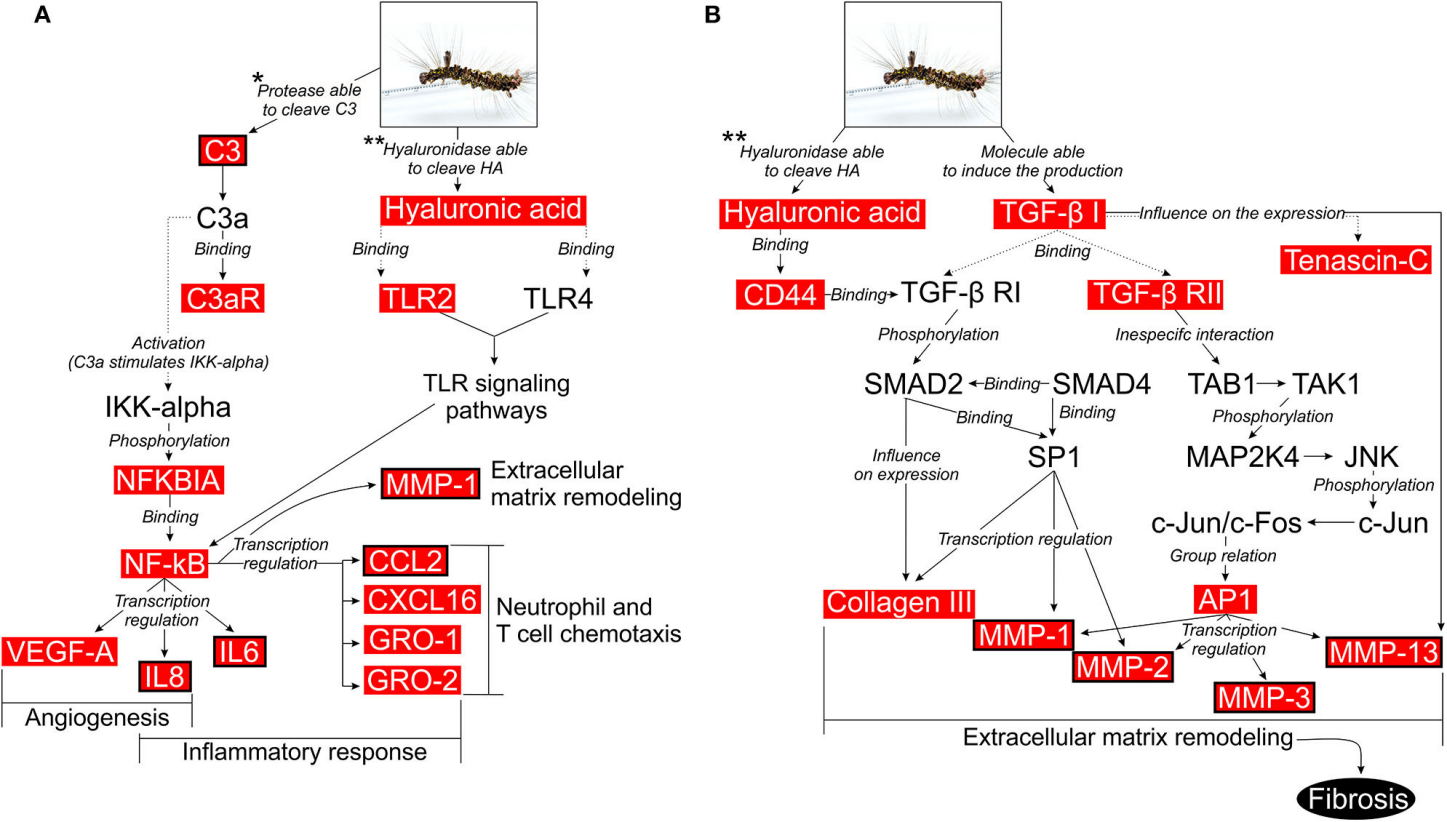
Possible intracellular signaling pathways activated in chondrocytes upon treatment with pararama hair extract. **(A)** Activation of cells by the complement system or by the interaction between low molecular weight hyaluronan and TLR2/TLR4. **(B)** Activation of cells by the interaction between low molecular weight hyaluronan and CD44 or by interaction between TGF-β receptors and TGF-β1. Illustration based on the data obtained with the use of the MetaCore pathway analysis tool (GeneGO/Thomson Reuters) and enriched with DEGs, which are highlighted in red boxes. Red boxes with black borders are the molecules that were validated in this study. Studies that assessed proteases that are able to cleave the C3 component and hyaluronidase that is able to cleave HA in the extract: (**7, *9).

We also performed a MetaCore enriched pathway (map) analysis to investigate the relationships between the DEGs and to find the most important signaling pathways in *P*. *semirufa* hair extract treatment (Extr × Ctrl). MetaCore identified 452 enriched pathways for Ext × Ctrl, of which 260 contained at least one of those selected genes. We selected 19 out of the 260 pathways associated with inflammation and OA (Supplementary Table 3), some of which will be further discussed here. This rationale led us to identify pathways associated with the immune response, angiogenesis, ECM remodeling, and release of proinflammatory mediators as the most significant signaling pathways associated with pararamosis pathogenesis (Figure 6).

## Discussion

Pararamosis is caused by accidental penetration of human subcutaneous tissue by *P*. *semirufa* caterpillar hairs. Over time, this condition can evolve into osteoarticular deformities due to impaired cartilage resulting from an inflammation of the joints, similar to other joint diseases. Understanding of the molecular mechanisms involved in pararamosis may contribute to establishing more effective therapeutic approaches for this occupational neglected disease, which affects communities such as the rubber tappers of the Amazon rainforest.

This study aimed to assess the effects of the *P*. *semirufa* hair extract on chondrocytes, an important cell type that is present in the joint and is involved in the onset of joint diseases. We treated these cells with IL-1β, as a positive control, since this is a well-known mediator that is involved in the pathophysiology of joint inflammation (28). We investigated a panel of cytokines, chemokines, MMPs, complement components, eicosanoids, and ECM components related to OA and RA that are potentially produced by chondrocytes in response to the pararama hair extract or IL-1β treatment. Another approach was the transcriptomic analysis of treated cells and the selection and testing of 46 genes involved in OA to verify whether the extract induced a disease with an OA signature.

The analyses of cytokines and chemokines in the supernatant of chondrocytes treated with the *P*. *semirufa* hair extract showed a time-dependent increase in the levels of IL-6, IL-8, and MCP-1 (Figure 1), which was confirmed by RT-qPCR experiments (Figure 5). IL-6 and CXCL8 (IL-8) are proinflammatory and angiogenic cytokines that are potent chemoattractants for neutrophils. Several studies have shown increased levels of IL-6 and IL-8 in peripheral blood mononuclear cells or the bone marrow of patients with rheumatoid arthritis (29). Moreover, IL-8 expression is associated with chondrocyte hypertrophy and cartilage destruction in osteoarthritis (30, 31). CCL2 (MCP-1) is a member of the β-chemokine family and, when produced at high levels, it regulates the immune process, triggers chemotaxis, activates macrophages and takes part in the activation of mast cells and the production of leukotrienes (32). Some authors have reported increased MCP-1 levels in the inflammatory process, both in RA and in OA (32, 33).

The extract also induced chondrocytes to produce PGE_2_ (Figure 1). In patients with OA, PGE_2_ expression is elevated, and it is associated with bone degeneration, cartilage metabolism, inhibition of proteoglycan biosynthesis, and joint pain (34–36). PGE_2_ is spontaneously released, and its production is induced by cyclooxygenase-2 (COX-2) expression (37). Thus, the extract may modulate chondrocytes to a proinflammatory profile similar to that found in patients with joint diseases, such as OA. In addition, the transcriptome analysis showed that the genes *PTGS2* (prostaglandin-endoperoxide synthase 2 or *COX-2*) and *PTGES* (prostaglandin E synthase) were highly upregulated in chondrocytes treated with the extract.

Additionally, we verified a time-dependent increase in MMP-1, MMP-2, MMP-3, and MMP-13 in the pararama hair extract-treated cultures (Figure 3), which was confirmed by RT-qPCR and transcriptome analyses, except for MMP-13 (Figure 5, Supplementary Table 2). MMPs play an important role in ECM turnover during embryogenesis, morphogenesis, normal tissue remodeling and repair, but in uncontrolled conditions, MMPs contribute to the pathogenesis of several diseases associated with tissue destruction, such as arthritis (38–42).

During OA progression, cytokines and chemokines such as IL-6, IL-8, MCP-1, and CCL5 (RANTES) actively participate in catabolic activities and are involved in cartilage destruction, such as through the production of MMP-1,−3 and−13 (28, 43–48). In addition, other proteinases produced by chondrocytes, such as MMP-2 and MMP-9, may also play a role in the degradation of several matrix components (49). Furthermore, IL-6 is an essential cytokine that triggers osteoclast differentiation and bone resorption (50, 51). Thus, increased release of cytokines and chemokines by chondrocytes treated with the pararama extract indicates a direct effect of extract component(s) on these cells, activating chondrocytes to produce cytokines and chemokines that may induce the production of MMPs by these same cells, which degrade matrix components. In addition, the downregulation observed in the *SOX9* gene following extract treatment (Figure 5) positively correlated with the downregulation of ECM transcription, since *SOX9* is responsible for the transcription of some ECM molecules, such as aggrecan and type II collagen (52).

Complement factors present in the synovial fluid originate from synovial cells, chondrocytes, infiltrating leukocyte or traumatic hemarthrosis (53–57). Analysis of complement components in the supernatants of chondrocyte cultures showed that C3, C4, and C5 were significantly higher in cells treated with the extract than in cells treated with buffer or IL-1β (Figure 2). This result suggests that the extract induces complement component production by direct action on chondrocytes or by indirect induction through cytokine production. Despite the increased production of C5, the concentration of this component in the supernatant decreased over time. As the extract contains serine proteases that are capable of cleaving complement components, including C5 (9), a reduction in this component may result from cleavage by the hair extract proteases.

During activation of the complement system, anaphylatoxins C3a and C5a are typical cleavage products that bind to their respective receptors C3aR and C5aR, expressed on a wide variety of cell types and induce inflammatory responses (58, 59). Nozaki et al. (60) identified these anaphylatoxins as proangiogenic factors that induce vascular endothelial growth factor (VEGF) expression in chorion tissue. Notably, VEGF expression has been observed during OA (61). Transcriptome analyses of chondrocytes treated with the extract and MetaCore analyses highlighted a complement activation pathway. Considering that C3 is a DEG and is present in the supernatants of chondrocyte cultures treated with the extract and that the extract proteases are able to directly cleave C3 and generate C3a (9), C3a fragments might bind to C3aR (also a DEG in our transcriptome analysis), activating nuclear factor kappa B (NF-κB) and the production of inflammatory cytokines, such as IL-6, and factors involved in angiogenesis, such as IL-8 and VEGF, thus resembling the events observed in joint diseases (Figure 6A).

Sequential events affect the homeostatic integrity of the extracellular matrix during OA progression, including a decrease in the amount of aggrecan and an increase in collagen (62–64). These changes also modify the collagen type composition from type II to type I, thereby affecting the mechanical stability of the extracellular matrix (65, 66). Results from the HCS experiments of chondrocyte cultures treated with the extract, confirmed by RT-qPCR (Figures 4, 5), suggest that the extract induces a reduction in aggrecan and type II collagen (I) by direct cleavage by extract proteases, (II) by inducing proteases expression, such as MMPs, by these cells, or (III) by inhibiting their gene expression. Transcriptome analysis showed that *ACAN (*aggrecan) was a highly downregulated DEG, while *COL2A1* was not a DEG but was a downregulated gene with low expression (Supplementary Table 2).

The protein high-mobility group box 1 (HMGB1) induces cytokine production and blood vessel formation and plays an important role in cell proliferation, differentiation, and migration (67). High levels of HMGB1 are observed in inflamed joints and serum of people with RA (68). HMGB1 was detected in chondrocyte nuclei, after 72 h of treatment with the extract (Figure 4), though both RT-qPCR, and the transcriptome analyses did not show any increase in *HMGB1* expression, perhaps due to differences in the treatment times used in the experiments.

Hyaluronic acid (HA), a polymer composed of glucuronic acid and N-acetyl glucosamine, is produced by hyaluronic acid synthases (HAS), expressed in fibroblast-like cells in the synovial lining and cartilage chondrocytes. HAS2 is the major isoform responsible for HA production in cartilage (69, 70). In chondrocytes, HA retains proteoglycans, such as aggrecan, and interweaves with collagen, providing a protective load-bearing surface (71, 72). Binding of HA to its primary receptor CD44 induces TGF-β receptor (TGFBR) activation, disturbances in cell adhesion to extracellular matrix components, inflammation, development, tumor growth, and metastasis (73, 74). RT-qPCR revealed an increase in *HAS2* expression and a reduction in *HYAL1, HYAL2*, and *HYAL3* expression after extract treatment (Figure 5). Transcriptome analysis showed an increase in the expression of *HAS2, HAS2-AS1* (anti-sense), *CD44*, and *TGFBR2*. We found a significant hyaluronidase activity in the hair extract (7), which may act on hyaluronic acid present in the chondrocyte ECM and activates the TGF-β pathway by increasing the HA-CD44-TGFBR interaction. TGF-β receptors interact with their ligand, TGF-β1, which is highly expressed in chondrocytes (75–77). We detected a slight increase in *TGF-*β*1* expression in treated cells (Supplementary Table 2). This interaction leads to the phosphorylation of SMAD2, which interacts with SMAD4. The SMAD2-SMAD4 complex translocates to the nucleus, where it modulates the transcription of TGF-β regulated genes, such as COL3A1 (Type III collagen) (Figure 6B). COL3A1 is a DEG that is highly expressed in chondrocytes treated with the extract, is related to the fibrosis process, and its expression is more pronounced in OA cartilage (78).

TGF-β receptors TGF-β1 interactions also activate MAP kinase pathways, such as the extracellular signal-regulated kinase (ERK) 1/2 pathway and the c-Jun N-terminal kinase (JNK) pathway. In the latter pathway, the transcription factor activator protein 1 (AP-1) is considered a key factor for MMP expression (79). MAP kinases are involved in MMP gene transcription (80), and in our study, they may be involved in the transcription of genes for matrix metalloproteinases such as MMP-1, MMP-2, MMP-3, and MMP-13 (Figure 6B). Tenascin-C (TNC), a hexameric glycoprotein component of the ECM, is a highly expressed molecule that participates in this pathway and was upregulated in extract treated chondrocytes. TNC interacts with over 25 different molecules, such as pathogenic components, matrix constituents, soluble factors, and cell surface proteins (81). TNC is a key molecule in tissue remodeling, and its deregulated increased expression is linked to joint diseases, including OA and RA. Thus, we again identified a proinflammatory profile induced by the extract in chondrocytes.

Low molecular weight hyaluronan (LMW-HA) is increased in joints in OA and has been shown to interact with TLR2/TLR4 in chondrocytes (82). In our model, this association also occurred in pararama hair extract treated chondrocytes. In fact, our transcriptomic analysis showed upregulation of the TLR2 in treated chondrocytes. This ligand and receptor interaction activates NF-κB, which is responsible for the transcription of chemokines *(IL-8, MCP-1, CXCL16, GRO1, and GRO2*), cytokines *(IL-6)*, and *MMP-1* by chondrocytes. These molecules contribute to chemotaxis, activation of inflammatory cells and ECM remodeling (Figure 6A). These factors were all highly expressed DEGs in chondrocytes that were treated with the extract.

In conclusion, our data shows that pararama hair extract induces chondrocyte inflammation, with the production of il-6, il-8, mcp-1, pge2, and complement components such as c3, c4, and c5. In addition, cartilage degradation and extracellular matrix remodeling features, such as increased expression of mmp1, mmp2, mmp3, and mmp13, and reduced type ii collagen and agrecan, were also observed. Transcriptomic and bioinformatics analyses of these cells indicated that the extract can activate important pathways related to the inflammatory process of joint diseases, such as the inflammatory response, chemotaxis of immune cells and extracellular matrix remodeling. Since the phenotype found in the human chondrocytes, treated with the extract, resembles those seen in joint diseases, such as oa, these data highlight the oa signature in pararamosis that should be further investigated in order to determine strategies to treat this and other joint diseases.

## Data Availability Statement

The datasets presented in this study can be found in online repositories. The names of the repository/repositories and accession number(s) can be found at: https://www.ncbi.nlm.nih.gov, PRJNA592966.

## Author Contributions

IV-B and DT designed research. IV-B, AC, CD-P, and KM performed the experiments. GP, IJ, AC-T, and DT contributed with biological material, reagents, and analytic tools. IV-B, FL, CD-P, CM, KM, and DT analyzed data. IV-B, FL, CM, and DT wrote the paper. All authors contributed to the article and approved the submitted version.

## Conflict of Interest

The authors declare that the research was conducted in the absence of any commercial or financial relationships that could be construed as a potential conflict of interest.
