# Supplementary material for: Human Chondrocyte Activation by Toxins From Premolis semirufa, an Amazon Rainforest Moth Caterpillar: Identifying an Osteoarthritis Signature
Source: Front Immunol. 2020 Sep 18;11:2191. doi: 10.3389/fimmu.2020.02191 (PMC7531038; doi:10.3389/fimmu.2020.02191)
Supplement: Supplementary file 1 [file Table_1.doc]

Supplementary Table 1. Sequence of primers used in RT-qPCR

| **Target** | **Primer** | **Sequence** |
| --- | --- | --- |
| *hMMP1* | Forward | AGTGACTGGGAAACCAGATGCTGA |
|  | Reverse | GCTCTTGGCAAATCTGGCGTGTAA |
| *hTGFβ1* | Forward | GAGCCTGGACACGCAGTACA |
|  | Reverse | CCCGGGTTATGCTGGTTGT |
| *hSOX9* | Forward | ACTCGCCACACTCCTCCTC |
|  | Reverse | CCCTCTCGCTTCAGGTCAG |
| *hP53* | Forward | AAACCACTGGCATGGAGAATATTTCA |
|  | Reverse | AACCATTTTCATGCTCTCTTTAACAA |
| *hIL10* | Forward | GAGCAGGTGAAGAATGCCTTTAA |
|  | Reverse | GATGTCAAACTCACTCATGGCTTT |
| *hIL1α* | Forward | ATTTGACATGGGTGCTTATAAGTCAT |
|  | Reverse | GGCAGTCACATACAATTGAGTTTTTG |
| *hHAS1* | Forward | CAAGGCGCTCGGAGATTC |
|  | Reverse | CCAACCTTGTGTCCGAGTCA |
| *hHAS2* | Forward | CAGACAGGCTGAGGACGACTTTAT |
|  | Reverse | GGATACATAGAAACCTCTCACAATGC |
| *hHAS3* | Forward | GGCGATTCGGTGGACTACAT |
|  | Reverse | CGATGGTGCAGGCTGGAT |
| *hHYAL1* | Forward | CGATGGTGCAGGCTGGAT |
|  | Reverse | GCCCCAGTGTAGTGTCCATATACTC |
| *hHYAL2* | Forward | GGCGCAGCTGGTGTCATC |
|  | Reverse | CCGTGTCAGGTAATCTTTGAGGTA |
| *hHYAL3* | Forward | TGTGCAGTCCATTGGTGTGA |
|  | Reverse | AAGGTGTCCACCAGGTAGTCATG |
| *hHMGB1* | Forward | AATACGAAAAGGATATTGCT |
|  | Reverse | GCGCTAGAACCAACTTAT |
| *hCOL1A* | Forward | AGAGCATGACCGATGGATTC |
|  | Reverse | TGTAGGCCACGCTGTTCTTG |
| *hACAN* | Forward | AAGACGGCTTCCACCAGTGT |
|  | Reverse | ATGCCATACGTCCTCACACC |
| *hBGN* | Forward | AGCTCCGCAAGGATGACTTC |
|  | Reverse | CCAGGTGGTTCTTGGAGATG |
| *hMMP3* | Forward | TGTTTTCTGGCCACAACTGC |
|  | Reverse | CTTGGGGTATCCGTGTAGCA |
| *hMMP13* | Forward | TGGTCCGATGTAACTCCTCTG |
|  | Reverse | CCCAGGAGGAAAAGCATGAG |
| hIL1β | Forward | CCATGGACAAGCTGAGGAAG |
|  | Reverse | ATCGTGCACATAAGCCTCGT |
| *hIL6* | Forward | TGGCTGAAAAAGATGGATGC |
|  | Reverse | CACAGCTCTGGCTTGTTCCT |
| *hIL8* | Forward | ACAAGAGCCAGGAAGAAACCA |
|  | Reverse | AGCACTCCTTGGCAAAACTG |
| *hIL18* | Forward | GGCAGATCACCAGAGGTCAG |
|  | Reverse | GGCATGCGTCACTACACTCA |
| *hTNFα* | Forward | CTCCCAGGTCCTCTTCAAGG |
|  | Reverse | GCTCTTGATGGCAGAGAGGA |
| *hHMBS** | Forward | TGGACCTGGTTGTTCACTCCTT |
|  | Reverse | CAACAGCATCATGAGGGTTTTC |
| *hHPRT** | Forward | TGAGGATTTGGAAAGGGTGT |
|  | Reverse | GAGCACACAGAGGGCTACAA |
| *hTUBA1C** | Forward | TCAACACCTTCTTCAGTGAAACG |
|  | Reverse | AGTGCCAGTGCGAACTTCATC |
| *hGAPDH** | Forward | TGCACCACCAACTGCTTAGC |
|  | Reverse | GGCATGGACTGTGGTCATGAG |
| *hACTB** | Forward | CGTCTTCCCCTCCATCGT |
|  | Reverse | GGGGTACTTCAGGGTGAGGA |

*Refers to the endogenous genes
