# Supplementary material for: Human Chondrocyte Activation by Toxins From Premolis semirufa, an Amazon Rainforest Moth Caterpillar: Identifying an Osteoarthritis Signature
Source: Front Immunol. 2020 Sep 18;11:2191. doi: 10.3389/fimmu.2020.02191 (PMC7531038; doi:10.3389/fimmu.2020.02191)
Supplement: Supplementary file 4 [file Data_Sheet_1.DOC]

Supplementary Material

**Supplementary Figures**

**Supplementary Figure 1. Pipeline diagram.** Quality and Quantification: We used FastQC and MultiQC to assess data quality and counted aligned reads using the Subread feature counts algorithm. Next, (i) we normalized the libraries and calculated DEGs using edgeR, and (ii) enrichment analysis was performed using MetaCore, which resulted in enriched pathways and their respective DEGs. We selected the most significant pathways related to inflammation and OA, and new heatmaps were plotted to identify whether they also clustered all three experimental groups well.

**Supplementary Figure 2. Heatmap and hierarchical clustering analysis of the expression of selected genes in human chondrocytes.** Rows correspond to all 46 selected genes, and the columns represent samples for each treatment: interleukin-1 beta (IL1B, colored light green), negative control (CTRL, colored blue) and Premolis semirufa hair extract treatment (EXT, colored dark green). The dendrogram discriminates all three groups well. Colors in the heatmap range from "Blue to Yellow to Red", with blue corresponding to downregulation and red corresponding to upregulation based on the RColorBrewer package.
