# Supplementary figures and images for: Human Chondrocyte Activation by Toxins From Premolis semirufa, an Amazon Rainforest Moth Caterpillar: Identifying an Osteoarthritis Signature
Source: Front Immunol. 2020 Sep 18;11:2191. doi: 10.3389/fimmu.2020.02191 (PMC7531038; doi:10.3389/fimmu.2020.02191)

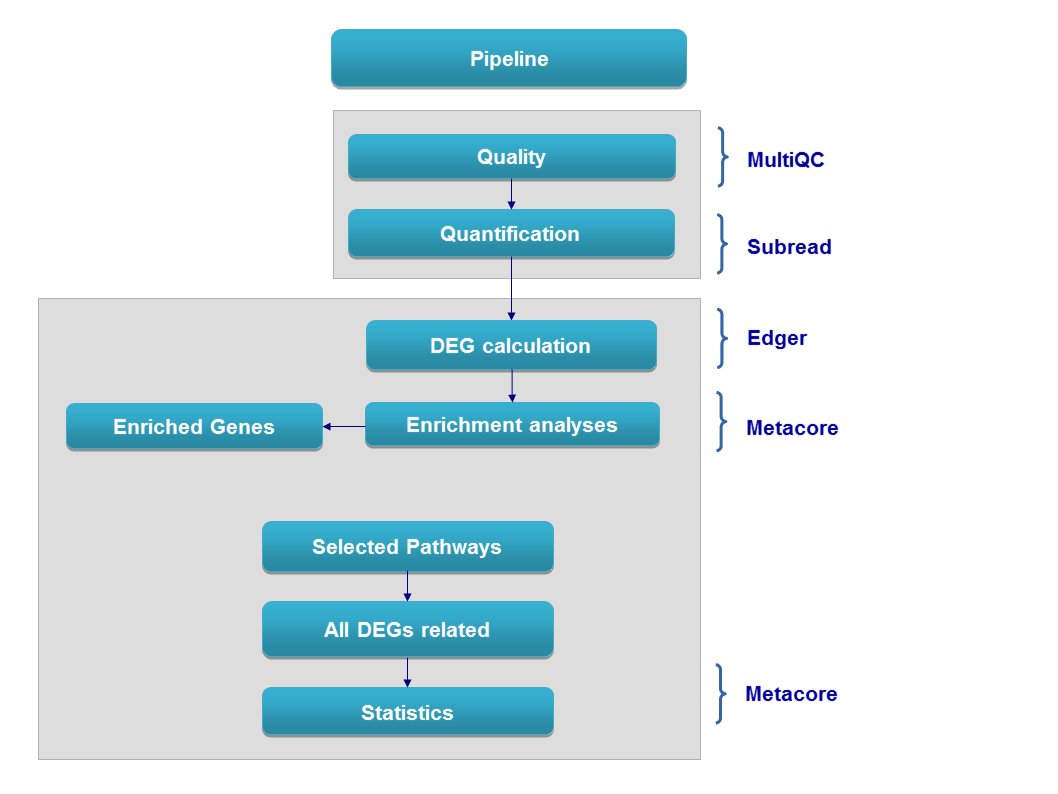

Supplement: Supplementary file 5 [file Image_1.TIF]

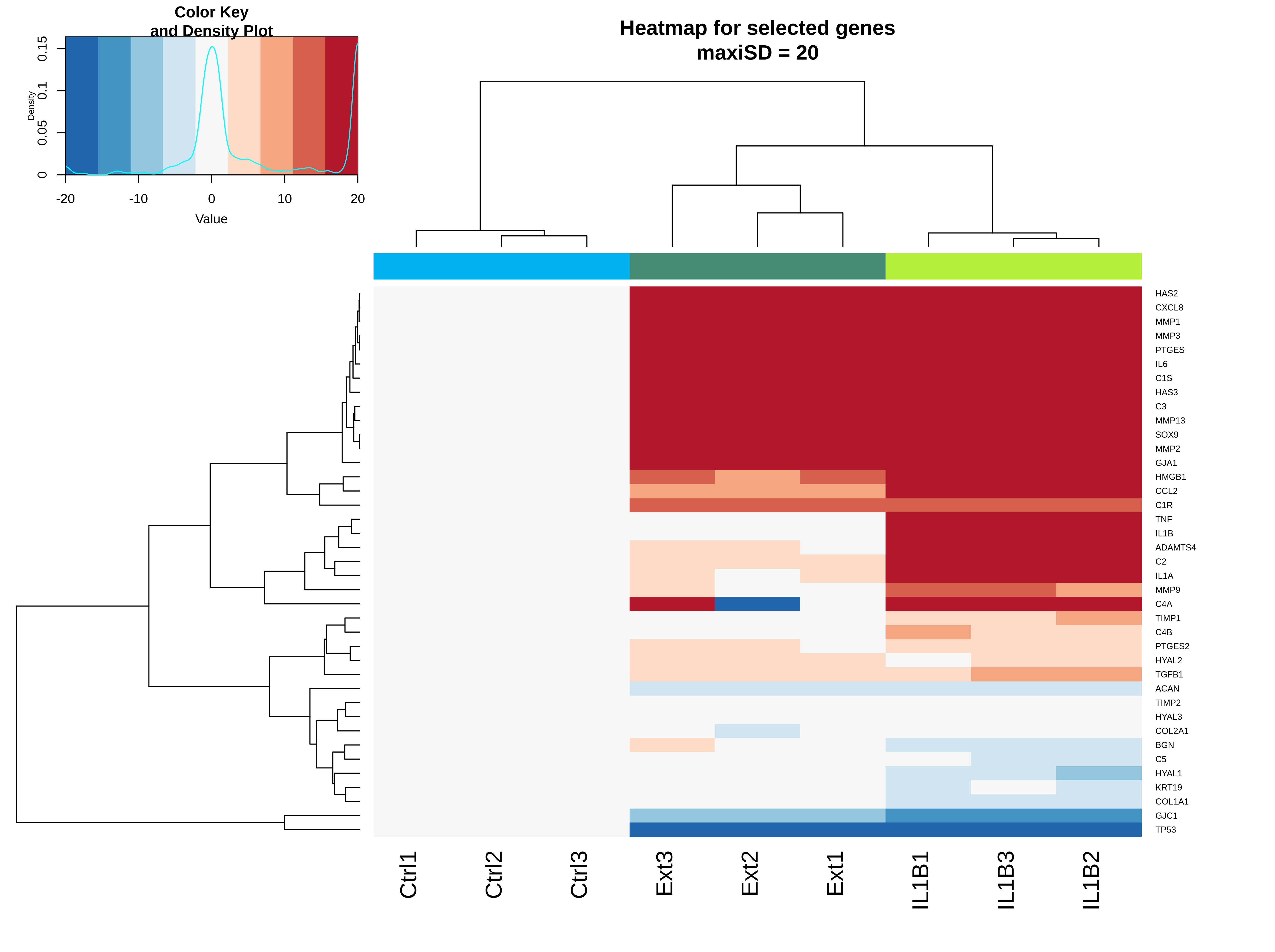

Supplement: Supplementary file 6 [file Image_2.TIF]
